# Supplementary material for: Deciphering hierarchical organization of topologically associated domains through change-point testing
Source: BMC Bioinformatics. 2021 Apr 10;22:183. doi: 10.1186/s12859-021-04113-8 (PMC8037919; doi:10.1186/s12859-021-04113-8)
Supplement: Supplementary file 1 — Additional file 1. Including the proof of Theorems 1 and 2, calculation of time complexity, Figs. S1 and S2, and Tables S1–S10. [file 12859_2021_4113_MOESM1_ESM.pdf]

Additional file 1: Decipher hierarchical organization of topologically associated domains through change-point testing

Haipeng Xing*[†], Yingru Wu[†], Michael Q. Zhang, Yong Chen*

1 Proof of Theorem 1 and 2

Theorem 1.

Before we prove Theorem 1, let's take a look at Gaussian distribution assumption. Instead of negative binomial we assume:

$$x_{ij} \sim N(\mu_k, \sigma^2), \quad 1 \le i \le j \le n, \quad \mu_k = \begin{cases} \mu_1, & \text{if}(i,j) \in A_{0,1} \\ \mu_2, & \text{if}(i,j) \in A_{1,2} \\ \mu_0, & \text{if}(i,j) \in R_{0,1,2} \end{cases}$$

Without loss of generality we take σ constant in the local region A, as they can be scaled to be equal. So we have log GLR test statistics:

$$GLR_{G,m} = \frac{1}{2\sigma^2}\Big(\frac{S_{A_1}^2}{|A_{0,1}|} + \frac{S_{A_2}^2}{|A_{1,2}|} + \frac{S_R^2}{|R|} - \frac{S_A^2}{|A|}\Big)$$

some algebra shows directly that $GLR_{G,m} = Z_m$.

Now we come back to negative binomial case.

$$\begin{aligned} GLR_{NB,m} = \sum_{k=1,2} \Big\{ & S_{A_k} \log\Big(\frac{S_{A_k}/|A_{k-1,k}|}{r + S_{A_k}/|A_{k-1,k}|}\Big) \\ & + r|A_{k-1,k}| \log\Big(\frac{r}{r + S_{A_k}/|A_{k-1,k}|}\Big)\Big\} \\ & + \Big(S_R \log\Big(\frac{S_R/|R|}{r + S_R/|R|}\Big) + r|R| \log\Big(\frac{r}{r + S_R/|R|}\Big)\Big) \\ & - \Big(S_A \log\Big(\frac{S_A/|A|}{r + S_A/|A|}\Big) + r|A| \log\Big(\frac{r}{r + S_A/|A|}\Big)\Big) \end{aligned} \quad (1.1)$$

*Correspondence: haipeng.xing@stonybrook.edu; chenyong@rowan.edu
[†]Equal contributor

Under null hypothesis, consider x_{ij} from region A in 1.1. By central limit theorem we have:

$$S_A/|A| = \frac{\phi r}{1-\phi} + O_p(|A|^{-1/2}) \tag{1.2}$$

where $E(S_A/|A|) = \frac{\phi r}{1-\phi}$. By Taylor expansion around the mean:

$$\begin{aligned}(S_A/|A|)\log\Big(\frac{S_A/|A|}{r+S_A/|A|}\Big) &= \frac{\phi r}{1-\phi}\log(\frac{\phi}{1-\phi}) \\ &+(1-\phi+\log\phi)\Big(S_A/|A| - \frac{\phi r}{1-\phi}\Big) \\ &+\frac{1}{2}\Big(\frac{(1-\phi)^2}{\phi r} - \frac{(1-\phi)^2}{r}\Big)\Big(S_A/|A| - \frac{\phi r}{1-\phi}\Big)^2 \\ &+o_p(|A|^{-1})\end{aligned}$$

$$\begin{aligned}\log\Big(\frac{r}{r+S_A/|A|}\Big) &= \log(1-\phi) - \frac{1-\phi}{r}\Big(S_A/|A| - \frac{\phi r}{1-\phi}\Big) \\ &+\frac{(1-\phi)^2}{2r^2}\Big(S_A/|A| - \frac{\phi r}{1-\phi}\Big)^2 + o_p(|A|^{-1}).\end{aligned}$$

So we have the last term in 1.1 as:

$$\begin{aligned}&S_A\log\Big(\frac{S_A/|A|}{r+S_A/|A|}\Big) + r|A|\log\Big(\frac{r}{r+S_A/|A|}\Big) \\ &= |A|(S_A/|A|)\log\Big(\frac{S_A/|A|}{r+S_A/|A|}\Big) + r|A|\log\Big(\frac{r}{r+S_A/|A|}\Big) \\ &= \frac{1}{2}\frac{(1-\phi)^2}{\phi r}\frac{S_A^2}{|A|} + c_1 S_A + c_2|A| + o_p(1)\end{aligned}$$

Where c_1 and c_2 are some constants. Similarly we do the same to other terms in 1.1. By the fact that $S_A = S_{A_1} + S_{A_2} + S_R$ and $|A| = |A_{0,1}| + |A_{1,2}| + |R|$, all the first order terms of S_A, S_{A_1}, S_{A_2}, S_R, $|A|$, $|A_{0,1}|$, $|A_{1,2}|$ and $|R|$ are cancelled.

Because m/n holds constant, $\frac{|A_{0,1}|}{|A|}$, $\frac{|A_{1,2}|}{|A|}$ and $\frac{|R|}{|A|}$ also hold constant. We have under the null hypothesis:

$$\frac{2\phi r}{(1-\phi)^2}GLR_{NB,m} = \frac{S_{A_1}^2}{|A_{0,1}|} + \frac{S_{A_2}^2}{|A_{1,2}|} + \frac{S_R^2}{|R|} - \frac{S_A^2}{|A|} + o_p(1) \tag{1.3}$$

$$GLR_{NB,m} = Z_m + o_p(1) \tag{1.4}$$

Notice that $\frac{\phi r}{(1-\phi)^2}$ is the variance of negative binomial distribution. So under null hypothesis, if we have all the elements scaled by the common σ_0 they have

variance equals to 1.

Last, if we assume read counts are Poisson random variables with blockwise constant parameter λ, the GLR test statistics is:

$$GLR_{P,m} = S_{A_1} \log \frac{S_{A_1}}{|A_{0,1}|} + S_{A_2} \log \frac{S_{A_2}}{|A_{1,2}|} + S_R \log \frac{S_R}{|R|} - S_A \log \frac{S_A}{|A|}$$

By exactly similar arguments we have under null hypothesis:

$$2\lambda GLR_{P,m} = \frac{S_{A_1}^2}{|A_{0,1}|} + \frac{S_{A_2}^2}{|A_{1,2}|} + \frac{S_R^2}{|R|} - \frac{S_A^2}{|A|} + o_p(1) \tag{1.5}$$

$$GLR_{P,m} = Z_m + o_p(1) \tag{1.6}$$

Therefore, The GLR statistics $GLR_{G,m}, GLR_{P,m}, GLR_{NB,m}$ are asymptotically equivalent to Z_m. □

Theorem 2.

Consider a Gaussian random field $G(s,t)$ defined on the upper triangular part of a unit square, $B = \{(s,t)|0 \leq s \leq t \leq 1\}$. The random field $G(s,t)$ satisfies the following properties: (1) for $s,t \in (0,1)$ and $s < t$, $\partial^2 G(s,t)/\partial s \partial t$ are normally distributed as $N(0, dtds)$; (2) for $t \in (0,1)$, $\partial^2 G(t,t)/\partial t^2$ are normally distributed as $N(0, \frac{1}{2}(dt)^2)$; (3) for regions $B_i \subset B$, $i = 1,2$, $\mathrm{Cov}(G(B_1), G(B_2)) = |B_1 \cap B_2|$. For region $\widetilde{B} \subset B$, we define the integral

$$G_{\widetilde{B}} = \int\int_{(s,t)\in\widetilde{B}} G(s,t)dsdt$$

It is obvious that, as $n \to \infty$,

$$\frac{|A_1|}{n^2} \to \frac{1}{2}t^2, \quad \frac{|A_1|}{|A_1 \cup R_t|} \to \frac{t}{2-t}, \quad \frac{|A_1 \cup R_t|}{|A|} \to t(2-t).$$

Then by Donsker's theorem:

$$\frac{S_{A_1}}{\sqrt{n^2/2}} \to G_{\widetilde{A}_1}, \quad \frac{S_{A_1 \cup R_t}}{\sqrt{n^2/2}} \to G_{\widetilde{A}_1 \cup \widetilde{R}_t}, \quad \frac{S_A}{\sqrt{n^2/2}} \to G_{\widetilde{A}}.$$

Hence,

$$Z_m \to g_t, \quad \widetilde{Z} \to g_\delta.$$

for $m_0/n \to \delta > 0$.

□

2 The simulated distribution of $\widetilde{Z}$

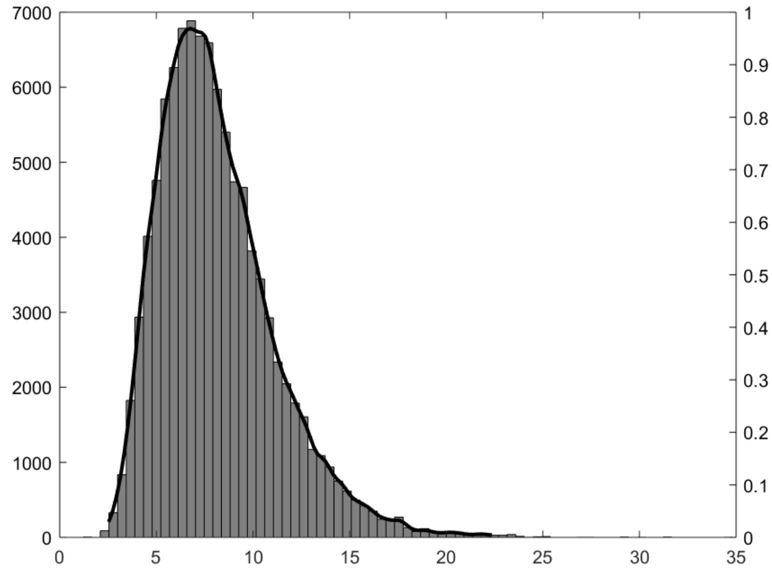

Fig. S1. Historgram and density function of simulated $\widetilde{Z}$ with $n = 10^5$ and $m_0 = n \cdot 2.5\%$.

3 Theoretical and practical time complexity

In the best case, we have $r = log_2 n$ iterations. The time complexity is as follows:

$$\big(2\times\frac{n^2+n}{2}+4n\big)+\big(2^2\times\frac{(n/2)^2+n/2}{2}+4n\big)+...+\big(2^r\times\frac{(n/2^{r-1})^2+n/2^{r-1}}{2}+4n\big)$$

$$= n^2\big(1+\frac{1}{2}+\frac{1}{4}+...\big)+5nr = O(n^2)$$

In the worst case, one of the two sub-matrices we divide is as small as possible with size ξ in each iteration. We have $r = n/\xi$ iterations.

$$\big(2\times\frac{n^2+n}{2}+4n\big)+\big(2\times\frac{(n-\xi)^2+(n-\xi)}{2}+4(n-\xi)\big)+...+\big(2\times\frac{(2\xi)^2+2\xi}{2}+4\times 2\xi\big)$$

$$= \big(n^2+(n-\xi)^2+...+(2\xi)^2\big)+5\big(n+(n-\xi)+...+2\xi\big) = O(n^3)$$

We also tested HiCKey on Chr1 matrices from GM12878 cell line. The following figure shows the real performance of running time and memory usage.

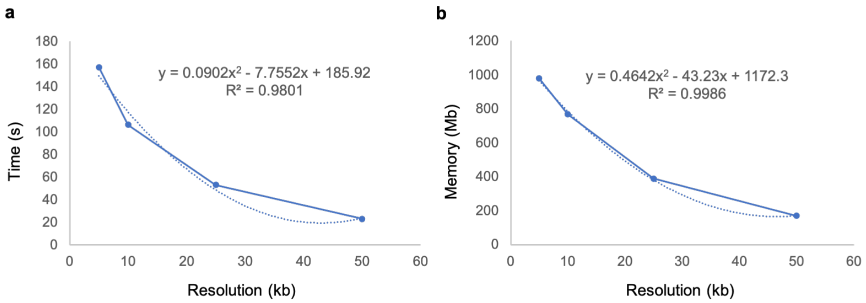

Fig. S2. The practical running time and memory usage. (**A**) Running time and resolution. (**B**) Memory and resolution. Both of the polynomial functions were calculated by Excel.

4 Performance of HiCKey and other methods in simulation datasets Sim1 and Sim2

Table S1. Performance of change-point estimation in Sim1 at different noise levels. The numbers in each cell show the mean and standard error.

		4%	8%	12%	16%
HiCKey	$\widehat{K}-K$	-0.2 (0.20)	1.8 (0.37)	19.4 (3.22)	82.6 (2.11)
	TPR	.9988 (.0012)	.9930 (.0022)	.9812 (.0039)	.9459 (.0087)
	FDR	.0000 (.0000)	.0173 (.0031)	.1176 (.0163)	.3618 (.0086)
IC-Finder	$\widehat{K}-K$	207.4 (10.78)	113.8 (4.68)	98.6 (4.13)	104.0 (2.21)
	TPR	.5593 (.0150)	.8430 (.0206)	.8779 (.0110)	.8930 (.0047)
	FDR	.7441 (.0148)	.4902 (.0163)	.4395 (.0090)	.4412 (.0069)
HiCSeg	$\widehat{K}-K$	-2.2 (0.37)	-29.2 (4.54)	-99.2 (9.57)	-150.2 (1.46)
	TPR	.9872 (.0022)	.8290 (.0256)	.4232 (.0557)	.1244 (.0087)
	FDR	.0000 (.0000)	.0013 (.0013)	.0000 (.0000)	.0182 (.0182)
TADTree	$\widehat{K}-K$	397.6 (35.33)	230 (28.55)	145.8 (27.83)	67.4 (15.49)
	TPR	.7791 (.0144)	.7826 (.0193)	.8035 (.0317)	.8465 (.0113)
	FDR	.8227 (.0156)	.7347 (.0258)	.6559 (.0321)	.5311 (.0306)
3DNetMod	$\widehat{K}-K$	519.40 (39.50)	558.80 (42.92)	424.60 (31.89)	349.00 (13.78)
	TPR	.9205 (.0156)	.9345 (.0130)	.8947 (.0188)	.8491 (.0093)
	FDR	.7695 (.0111)	.7788 (.0097)	.7413 (.0088)	.7200 (.0075)

Table S2. Performance of change-point estimation in Sim2 at different noise levels. The numbers in each cell show the mean and standard error.

		4%	8%	12%	16%
HiCKey	TPR	.8450 (.0070)	.7570 (.0090)	.6568 (.0044)	.5289 (.0118)
	FDR	.0590 (.0050)	.0867 (.0058)	.1343 (.0043)	.1928 (.0066)
IC-Finder	TPR	.5508 (.0075)	.7010 (.0082)	.6858 (.0172)	.6533 (.0117)
	FDR	.5934 (.0119)	.3111 (.0093)	.2674 (.0156)	.2860 (.0192)
HiCSeg	TPR	.5071 (.0089)	.0990 (.0016)	.0350 (.0009)	.0258 (.0015)
	FDR	.0000 (.0000)	.0144 (.0094)	.0268 (.0165)	.0400 (.0400)
TADTree	TPR	.6990 (.0074)	.7071 (.0097)	.6756 (.0099)	.5467 (.0497)
	FDR	.5334 (.0166)	.4938 (.0110)	.4494 (.0169)	.4268 (.0176)
3DNetMod	TPR	.6015 (.0102)	.4748 (.0165)	.3522 (.0098)	.2738 (.0083)
	FDR	.5879 (.0083)	.5228 (.0062)	.5077 (.0102)	.5701 (.0029)

5 Validating the performance of detecting hierarchical TAD structure in Sim2

Table S3. The upper panel is hierarchical similarity of HiCKey TADs and true structure in Sim2. The Fowlkes-Mallows indices (B_k) were calculated for four noise levels. The numbers in each cell show the mean and standard error. The lower panel is mean and standard deviation of B_k between the true structure and unrelated randomly relabeled nodes (Relabeling).

Method	B_k	4%	8%	12%	16%
HiCKey	B_1	.9242 (.0025)	.8676 (.0056)	.7784 (.0044)	.6887 (.0053)
	B_2	.9051 (.0071)	.8852 (.0045)	.8647 (.0103)	.8403 (.0063)
	B_3	.8196 (.0066)	.8168 (.0089)	.8148 (.0062)	.7702 (.0076)
Relabeling	B_1	.0030 (.0003)	.0031 (.0003)	.0034 (.0003)	.0040 (.0003)
	B_2	.0039 (.0003)	.0040 (.0003)	.0045 (.0003)	.0053 (.0003)
	B_3	.0051 (.0003)	.0054 (.0003)	.0058 (.0003)	.0072 (.0004)

6 Robustness of HiCKey against different distributions of HiC data

Table S4. Robustness against different distributions. The numbers in each cell show the mean and standard error.

Setting	Noise	$\hat{K}-K$	TPR	FDR
Sim3	0%	0.15(0.02)	1(0)	.0046(4e-4)
	5%	0.13(0.01)	1(0)	.0041(4e-4)
	10%	0.15(0.01)	1(0)	.0047(4e-4)
	15%	0.13(0.01)	.9998(9e-5)	.0043(4e-4)
Sim4	0%	0.14(0.01)	.9999(7e-5)	.0045(4e-4)
	5%	0.16(0.01)	.9998(9e-5)	.0050(4e-4)
	10%	0.15(0.01)	.9998(7e-5)	.0048(4e-4)
	15%	0.14(0.01)	.9998(7e-5)	.0055(4e-4)

7 Robustness of HiCKey against decision in the first iteration

Table S5. Robustness against the first iteration in Sim1. The numbers in each cell show the mean and standard error.

	4%	8%	12%	16%
$\hat{K}-K$	0.06(0.0095)	0.02(0.0101)	0.72(0.0328)	-1.05(0.0421)
TPR	.9977(.0001)	.9846(.0002)	.9761(.0004)	.9745(.0003)

Table S6. Robustness against the first iteration in Sim2. The numbers in each cell show the mean and standard error.

	4%	8%	12%	16%
$\hat{K}-K$	0.58(0.0272)	0.27(0.0300)	-0.93(0.0607)	-0.87(0.0866)
TPR	.9950(.0001)	.9804(.0002)	.93825(.0005)	.9073(.0010)

Table S7. Robustness against the first iteration in hESC. The numbers in each cell show the mean and standard error.

Chr	$\hat{K} - K$	TPR
chr1	0.42(0.0525)	.96(.0003)
chr2	0.74(0.0664)	.94(.0013)
chr3	-0.66(0.0474)	.96(.0006)
chr4	-0.68(0.0595)	.94(.0006)
chr5	1.21(0.0465)	.96(.0006)
chr6	1.91(0.0794)	.94(.0012)
chr7	-1.35(0.0478)	.96(.0006)
chr8	-0.24(0.0351)	.96(.0009)
chr9	-0.85(0.0648)	.97(.0006)
chr10	1.33(0.0632)	.95(.0009)
chr11	0.06(0.0367)	.95(.0009)
chr12	-0.38(0.0560)	.95(.0006)
chr13	0.14(0.0569)	.93(.0012)
chr14	0.13(0.0465)	.95(.0009)
chr15	1.65(0.0402)	.95(.0009)
chr16	-0.08(0.0345)	.97(.0006)
chr17	0.10(0.0323)	.95(.0006)
chr18	-0.53(0.0373)	.93(.0012)
chr19	0.21(0.0402)	.90(.0009)
chr20	0.02(0.0259)	.94(.0009)
chr21	-0.26(0.0351)	.92(.0019)
chr22	-0.22(0.0332)	.95(.0012)
chr23	-1.52(0.0876)	.94(.0012)

Table S8. Robustness against the first iteration in IMR90. The numbers in each cell show the mean and standard error.

chr	$\hat{K} - K$	TPR
chr1	0.39(0.0519)	.97(.0003)
chr2	0.16(0.0531)	.95(.0006)
chr3	-1.44(0.0515)	.95(.0006)
chr4	-0.28(0.0572)	.95(.0006)
chr5	0.68(0.0557)	.96(.0006)
chr6	-2.09(0.0515)	.94(.0009)
chr7	-0.72(0.0351)	.97(.0003)
chr8	0.51(0.0376)	.96(.0006)
chr9	-0.49(0.0379)	.96(.0006)
chr10	-0.25(0.03826)	.96(.0006)
chr11	0.01(0.0386)	.97(.0006)
chr12	0.30(0.0402)	.96(.0006)
chr13	1.26(0.0468)	.93(.0013)
chr14	1.45(0.0424)	.94(.0012)
chr15	-0.37(0.0427)	.97(.0006)
chr16	-0.07(0.0503)	.96(.0006)
chr17	-2.50(0.0458)	.93(.0012)
chr18	-0.04(0.0326)	.96(.0006)
chr19	-0.33(0.0335)	.92(.0012)
chr20	-0.36(0.0300)	.94(.0009)
chr21	-0.18(0.0294)	.91(.0016)
chr22	-0.90(0.0490)	.94(.0012)
chr23	-0.19(0.0538)	.96(.0006)

8 The statistical result of hierarchical orders in 7 cell lines

Table S9. TAD number and percentage in each hierarchical layer.

Cell line	#TADs	order 1	order 2	order 3	≥order 4
GM12878	8586	7743 (90.18%)	687 (8.00%)	96 (1.12%)	14 (0.16%)
HMEC	8200	7072 (86.24%)	869 (10.60%)	183 (2.23%)	31 (0.38%)
HUVEC	8903	8053 (90.45%)	690 (7.75%)	98 (1.10%)	16 (0.18%)
IMR90	9043	8202 (90.70%)	689 (7.62%)	94 (1.04%)	12 (0.13%)
K562	8801	8046 (91.42%)	618 (7.02%)	87 (0.99%)	4 (0.05%)
KBM7	6246	5402 (86.49%)	647 (10.36%)	131 (2.10%)	21 (0.34%)
NHEK	7726	6935 (89.76%)	652 (8.44%)	87 (1.13%)	6 (0.08%)

9 TAD boundary enrichment in active chromosomal regions of 6 cell lines

Table S10. Number of bins and HiCKey boundaries in active and repressive regions. The enrichment of TAD boundaries is calculated by one-sided Fisher's exact test (p-value).

Cell Line	State	Bins	Boundaries	p-value
GM12878	active	29293	2490	5.15e-18
	repressive	81220	5553	
HESC	active	55345	4540	7.92e-4
	repressive	13010	950	
HUVEC	active	54380	4420	4.48e-12
	repressive	53832	3739	
IMR90	active	64220	5494	2.98e-58
	repressive	46400	2706	
K562	active	20960	1781	3.50e-9
	repressive	89932	6488	
KBM7	active	34269	2090	4.83e-15
	repressive	75936	3716	
